# m7G-related genes predict prognosis and affect the immune microenvironment and drug sensitivity in osteosarcoma

**DOI:** 10.3389/fphar.2023.1158775

**Published:** 2023-08-16

**Authors:** Zili Lin, Ziyi Wu, Yuhao Yuan, Wei Zhong, Wei Luo

**Affiliations:** ^1^ Department of Orthopaedics, Xiangya Hospital, Central South University, Changsha, China; ^2^ National Clinical Research Center for Geriatric Disorders, Xiangya Hospital, Changsha, China; ^3^ Department of Orthopaedics, The Second Xiangya Hospital, Central South University, Changsha, China

**Keywords:** m7G, immune infiltration, prognosis, osteosarcoma, AZD2014, drug sensitivity

## Abstract

**Background:** Osteosarcoma (OS), a primary malignant bone tumor, confronts therapeutic challenges rooted in multidrug resistance. Comprehensive understanding of disease occurrence and progression is imperative for advancing treatment strategies. m7G modification, an emerging post-transcriptional modification implicated in various diseases, may provide new insights to explore OS pathogenesis and progression.

**Methods:** The m7G-related molecular landscape in OS was probed using diverse bioinformatics analyses, encompassing LASSO Cox regression, immune infiltration assessment, and drug sensitivity analysis. Furthermore, the therapeutic potential of AZD2014 for OS was investigated through cell apoptosis and cycle assays. Eventually, multivariate Cox analysis and experimental validations, were conducted to investigate the independent prognostic m7G-related genes.

**Results:** A comprehensive m7G-related risk model incorporating eight signatures was established, with corresponding risk scores correlated with immune infiltration and drug sensitivity. Drug sensitivity analysis spotlighted AZD2014 as a potential therapeutic candidate for OS. Subsequent experiments corroborated AZD2014's capability to induce G1-phase cell cycle arrest and apoptosis in OS cells. Ultimately, multivariate Cox regression analysis unveiled the independent prognostic importance of CYFIP1 and EIF4A1, differential expressions of which were validated at histological and cytological levels.

**Conclusion:** This study furnishes a profound understanding of the contribution of m7G-related genes to the pathogenesis of OS. The discerned therapeutic potential of AZD2014, in conjunction with the identification of CYFIP1 and EIF4A1 as independent risk factors, opens novel vistas for the treatment of OS.

## Introduction

Osteosarcoma (OS) is the most commonly diagnosed malignant bone tumor characterized by the presence of intratumoral osteogenesis (Wylie, 2004). Its annual incidence in the general population ranges from two to three cases per 1,000,000 individuals, but notably peaks at eight to eleven cases per 1,000,000 individuals among children and adolescents (Ritter and Bielack, 2010). However, the introduction of multi-agent chemotherapy has improved the 5-year overall survival rate for OS, achieving approximately 60%–70% long-term survival (Gill and Gorlick, 2021), and the prognosis for patients with metastatic or relapsed disease remains devastating, with a mere 5-year overall survival rate of 20% (Link et al., 1986; Aljubran et al., 2009; Meyers et al., 2011). Moreover, the emergence of chemotherapy resistance in OS has hindered therapeutic advancements over the past decades (Gill and Gorlick, 2021). Molecularly, several crucial factors contribute to the suboptimal outcomes in OS treatment. First, the lack of sensitive markers for subtyping patients with poor prognosis hampers personalized treatment strategies. Second, the intricate immunological microenvironment of OS remains poorly understood, limiting our ability to harness immune-based therapies effectively. Last, the complex genetic landscape of OS presents challenges in identifying and targeting key molecular drivers for therapeutic intervention (Meltzer and Helman, 2021). Consequently, there is a pressing need to gain novel insights into the molecular genetics of OS as such discoveries hold immense potential in optimizing early detection methods, advancing treatment modalities, and enhancing prognostic predictions for OS patients.

N7-methylguanosine (m7G) is a significant post-transcriptional modification, which means that a methyl group is added to the seventh N position of RNA guanine by methylation transferase (Guy and Phizicky, 2014; Sloan et al., 2017; Song et al., 2020). Through the methylation modification of RNA, m7G influences the production, maturation, and decomposition of RNA by triggering various biological and pathological reactions (Song et al., 2020). Recently, increasing evidence has shown that m7G modification involves the oncogenesis and progression of various cancers and plays the role of a double-edged sword (Chen Y. et al., 2022; Luo et al., 2022). For example, m7G-related genes serve as tumor promoters in various cancers, such as glioma, hepatocarcinoma, esophageal squamous cell carcinoma, non-small-cell lung cancer, and head and neck squamous cell carcinoma, but exhibit an anti-tumor effect in certain cancers (Pandolfini et al., 2019; Tian et al., 2019; Chen et al., 2021; Li et al., 2021; Chen J. et al., 2022; Han et al., 2022). Recently, increasing research focused on the role of m7G in the tumor microenvironment (TME) (Huang et al., 2022a; Li Z. et al., 2022; Gao et al., 2022; Zeng et al., 2023). Certain studies have exhibited that m7G-related genes shape the TME by affecting the distribution of immune cells (Wang et al., 2017; Chen J. et al., 2022; Dong et al., 2022; Wu et al., 2022). Furthermore, studies have found that m7G was associated with response to chemotherapeutic drugs, including cisplatin and docetaxel (Okamoto et al., 2014). As such, exploring the mechanism of m7G during the occurrence and development of OS may promote the advancement of OS treatment.

Herein, we utilized the bioinformatic analysis to explore the role of m7G-related genes in the molecular landscape of OS. Additionally, we constructed a prognostic model and its relation with immune infiltration and chemotherapy sensitivity. Furthermore, an integrated nomogram was established to quantitatively predict OS patients’ prognosis. More importantly, through drug sensitivity analysis, we found that AZD2014 may serve as the potential sensitive drug for OS, and AZD2014 can induce the G1-phase cell cycle arrest and apoptosis of OS cells, serving as a potential therapeutic drug for OS patients. Eventually, we identified CYFIP1 and EIF4A1 as two independent risk factors of OS through multivariate Cox regression analysis and verified the differential expression of CYFIP1 and EIF4A1 at histological and cytological levels, offering new therapeutic targets for OS.

## Materials and methods

### Data collection and processing

The RNA sequencing data of OS patients with corresponding clinical information in TARGET-OS and GSE21257 datasets were separately downloaded from Therapeutically Applicable Research to Generate Effective Treatments (TARGET; https://ocg.cancer.gov/programs/target) and the Gene Expression Omnibus (GEO; https://www.ncbi.nlm.nih.gov/geo/) databases. Additionally, the mRNA expressions of OS patients in these two cohorts were merged and batch-corrected via the “sva” package. The m7G-related genes were collected based on the published literature (Regmi et al., 2022). Detailed information on the 98 m7G-related genes is shown in Supplementary Table S1.

### Screening of prognostic m7G-related genes and unsupervised consensus clustering

Univariate Cox regression analysis was performed to identify the prognostic m7G-related genes of TARGET-OS patients. The “NMF” package was applied to identify different m7G-related clusters based on the expressions of prognostic m7G-related genes. The “ns” algorithm was used as a clustering measure, and the cophenetic coefficient was applied to decide the best clustering. After the best cophenetic coefficients were selected, a heatmap was depicted using the “consensusmap” function. The “limma” package was adopted to determine differentially expressed genes (DEGs) among different m7G-related clusters with a Foldchange >1.5 and *p*-value <0.05.

### Construction and verification of a prognostic model based on m7G-related genes

The TARGET-OS dataset was used as the training cohort to construct a prognostic model**.** The least absolute shrinkage and selection operator (LASSO) analysis was used to further narrow the preliminarily screened prognostic m7G-related genes. The final prognostic model can be expressed as follows: risk score = ∑Coef_mRNAi_ * Expression_mRNAi_, in which Expression_mRNAi_ represents the expression level of each prognostic m7G-related gene and Coef_mRNAi_ represents the coefficient of the corresponding prognostic m7G-related genes in the LASSO-Cox regression model. Based on the prognostic model, the risk score of each OS patient was calculated, and OS patients were separated into high- and low-risk groups based on the medium risk score. In addition, OS patients included in the GSE21257 dataset were set as a verification cohort to validate the prediction performance of the constructed prognostic model. Furthermore, the prediction performance of the constructed prognostic model was validated in the merged dataset.

### Construction of an integrated nomogram

We utilized the “survival,” “survminer,” and “rms” packages in R to develop an integrated nomogram. Multivariate Cox regression models were performed to identify the independent factors associated with the prognosis of OS patients. Following the result of multivariate analysis, a Cox proportional hazard (PH) model was applied to develop a nomogram to predict the survival probability of OS patients of the merged dataset. The receiver operating characteristic (ROC) curve, calibration curve, and decision curve analysis (DCA) curve were performed to assess the predictive accuracy and clinical usefulness.

### Functional analyses and mechanism exploration

The underlying effect of m7G-related genes on the occurrence and development of OS was investigated through functional enrichment analysis. First, DEGs between the merged datasets’ high- and low-risk groups were identified with the R package “limma.” Subsequently, the functional enrichment analyses, including Gene Ontology (GO) and Kyoto Encyclopedia of Genes and Genomes (KEGG) analyses, were completed to explore underlying pathways. In addition, a protein–protein interaction (PPI) network based on identified DEGs was constructed on the Metascape website (http://metascape.org/gp/index.html) to screen hub genes and hub modules. Subsequently, the Gene Set Enrichment Analysis (GSEA) algorithm was applied to explore the activity variation of KEGG analysis. Moreover, the GSVA algorithm was applied to explore the activity variation of biological process (BP) terms in GO analysis.

### Immune infiltration and drug sensitivity analysis

The Estimation of Stromal and Immune cells in Malignant Tumor tissues using Expression data (ESTIMATE) algorithm was performed to calculate the stromal score, immune score, ESTIMA score, and tumor purity in the high- and low-risk groups. Moreover, MCP counter algorithms were used to estimate the proportion of immune cells. The Genomics of Drug Sensitivity in Cancer (GDSC), developed by the Sanger Institute in the UK, is considered the most prominent public resource for drug sensitivity of tumor cells at present, collecting the sensitivity and response of tumor cells against drugs. The “limma,” “ggpubr,” and “oncopredict” packages were utilized to perform drug sensitivity between the high- and low-risk groups in the merged dataset and to screen potential therapeutic drugs for OS, with *p* < 0.01 as the screening criterion.

### Tissue, cell lines, and reagents

OS samples and adjacent normal tissues were collected from patients undergoing hinge knee arthroplasty in the Orthopedics Department of Xiangya Hospital. The Ethics Committee of Xiangya Hospital of Central South University approved this study, and informed consent was obtained from all the participants or their legal guardians.

The human OS cell lines U2OS and MG63 cells were purchased from the Procell Life Science&Technology Company. Under 37°C and 5% CO_2_, U2OS cells were maintained in high-glucose DMEM, while MG63 cells were cultured in MEM, and all mediums were added with 10% fetal bovine serum and 1% streptomycin/penicillin.

AZD2014 was purchased from the Selleck Company (Houston, TX, USA). Dimethyl sulfoxide (DMSO) was utilized to dissolve the AZD2014 powder to prepare a 50 mM stock solution stored at −80°C. The stock solution was diluted with the appropriate assay medium in the subsequent experiment, while 0.1% DMSO was used as the vehicle control.

### Immunohistochemical analysis

The pathological tissues and adjacent normal tissues of OS patients were fixed in 4% paraformaldehyde, embedded in paraffin, and then, sliced into slides for immunohistochemistry (IHC). Subsequently, deparaffinization, dehydration, and antigen reparation were performed for each slide. To block the endogenous peroxidase activity, the slides were incubated with 3% hydrogen peroxide solution at room temperature for 10 min. After rinsing with PBS, the slides were hatched for 1 hour at room temperature with the goat serum (ZLI-9022, ZSGB-Bio, China). Then, the slides were hatched with *EIF4A1* primary antibody (R383037, ZenBio, China) and *CYFIP1* primary antibody (ab156016), which were diluted into 1:100, respectively, at room temperature overnight. After rinsing in PBS for three cycles for 5 min/times, the slides were hatched with an antibody booster and anti-rabbit secondary antibody (PV-9000, ZSGB-Bio, China) for 20 min at room temperature, respectively. Finally, the signals of sections were developed using 3,3′-diaminobenzidine tetrahydrochloride, and all slides were stained with hematoxylin.

### RNA extraction and quantitative PCR

Total RNA was extracted from hFOB1.19, U2OS, and MG63 cell lines by using the AG RNAex Pro RNA extraction kit (AG, Changsha, China) and utilized to synthesize cDNA with the Reverse Transcription Kit (AG, Changsha, China). qPCR analysis was carried out on the ABI7500 system using the TB Green Premix Pro Taq HS qPCR Kit (AG, Changsha, China). Last, we used the ΔΔCq method to calculate the relative expression levels of each sample, and the results were expressed as 2^−ΔΔCq^. GAPDH were used for normalization in the qPCR experiment.

### Cell cycle propidium iodide staining assay

For the cell cycle assay, cells were seeded in the six-well plates (5 × 10^5^/well) overnight and then treated with AZD2014 at the concentrations of 2.5, 5.0, and 10.0 uM. After 24 h, cells were collected and rinsed in PBS. We then fixed these samples in 70% ethanol overnight at 4°C. Subsequently, 0.5% propidium iodide (PI) (Multisciences Biotech Co., Ltd.) added with 0.01% RNase was used for staining. Cell cycle analysis was performed on the flow cytometer (NovoCyte, ACEA).

### Cell apoptosis assay

For the apoptosis assay, cells were seeded in six-well plates (2 × 10^5^/well) with AZD2014 at the concentrations of 2.5, 5.0, and 10 uM for 48 h. DMSO was used to treat the vehicle control group. The cells and supernatants were collected and washed twice with ice-cold PBS. The annexin V/PI kit (Multisciences Biotech Co., Ltd.) was used to detect apoptosis, according to the manufacturer’s instructions.

### Statistical analysis

Statistical tests were performed using R software (version 4.2.1). Continuous data were expressed as the mean and standard deviation, while categorical data were expressed as count and percentage. Univariate, LASSO, and multivariate Cox analyses were performed to identify independent prognostic factors and construct an integrated nomogram, including predictable clinical traitors and risk scores. The performance and clinical usefulness of the model were assessed by the calibration curve, timeROC, and timeDCA. All tests were two sided. The statistical significance was shown as follows: *p*-value <0.05 (*), *p*-value <0.01 (**), and *p*-value <0.001 (***).

## Results

### Identification of prognostic m7G-related genes and m7G-related clusters

**FIGURE 1:**
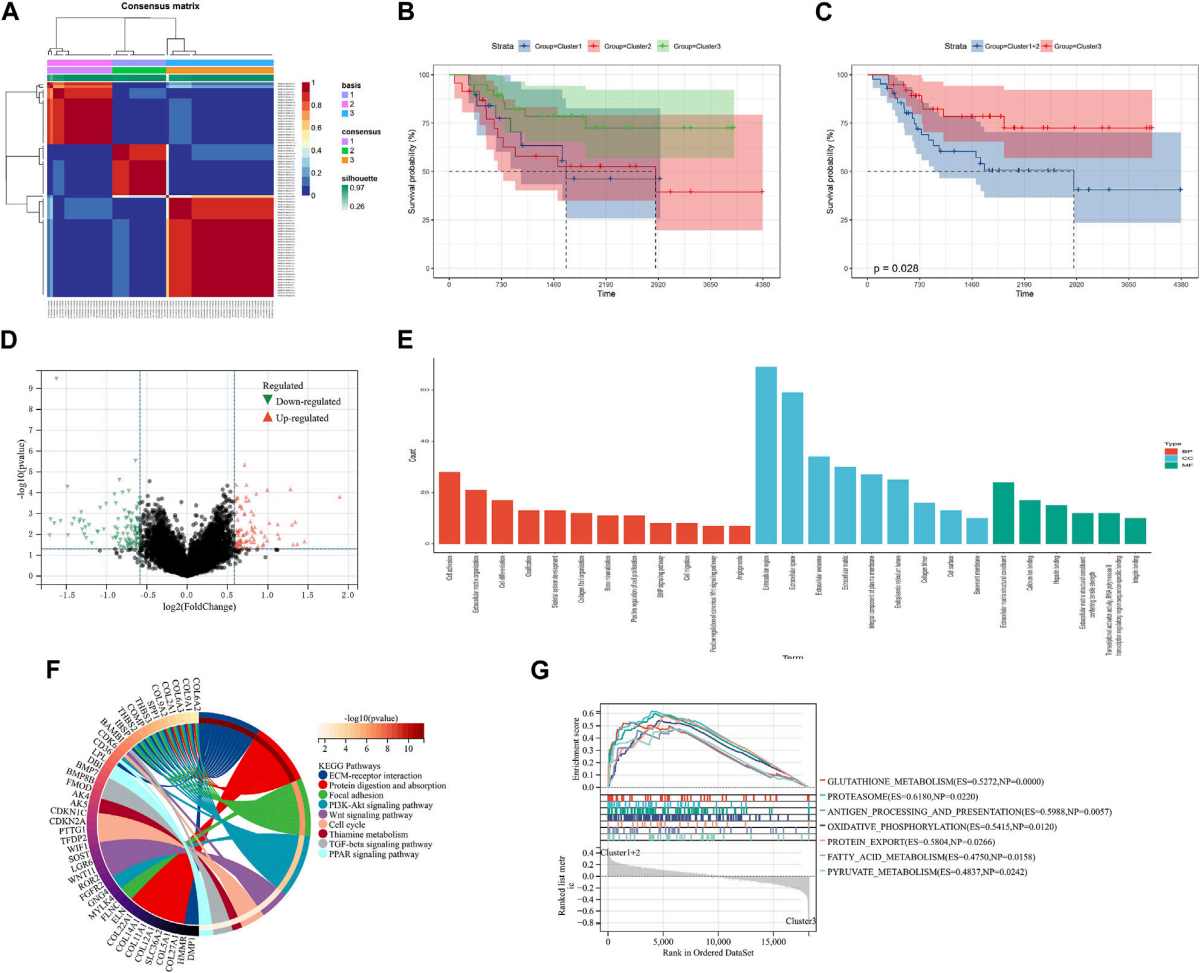
Screening of molecular subgroups. **(A)** Three clusters were identified as the optimal value for consensus clustering. **(B)** Kaplan–Meier plot of three clusters. **(C)** Kaplan–Meier plot of two new clusters. **(D)** DEGs between two new clusters. **(E)** GO analysis of DEGs. **(F)** KEGG analysis of DEGs. **(G)** GSEA of two new clusters. DEGs: differentially expressed genes; GO: Gene Ontology; KEGG: Kyoto Encyclopedia of Genes and Genomes; and GSEA: Gene Set Enrichment Analysis.

Ninety-eight m7G-related genes were obtained from the previous literature (Supplementary Table S1), and nine m7G-related genes were identified as the prognosis-related genes through univariate cox regression analysis: *CYFIP2*, *IGF2BP2*, *ALKBH1*, *NUDT1*, *FTO*, *EIF4A1*, *EIF4E3*, *NUDT16*, and *CYFIP1* (all *p* < 0.05) (Table 1). Based on the expression of nine identified m7G-related genes, we classified the patients into clusters to explore the impact of m7G RNA modification on OS patients. The cophenetic plot signified that dividing the patients into three clusters is the best clustering choice (Figure 1A; Supplementary Figure S1). Then, we used the Kaplan–Meier curve for these three clusters and found that the prognosis of Cluster 3 was better than that of Cluster 1 and Cluster 2 (Figure 1B, total *p*-value: 0.056, total HR: 0.65, 95CI% [0.41, 1.02]; Cluster1–Cluster2: *p*-value 0.91; Cluster1–Cluster3: *p*-value 0.08; and Cluster1–Cluster3: *p*-value 0.04). Subsequently, considering the similar prognoses between Cluster 1 and Cluster 2, we merged them into one group, plotted the Kaplan–Meier curve for the new clusters, and found the distinguished prognosis between these two clusters (*p*-value: 0.028, HR: 0.41, 95CI% [0.19, 0.93]) (Figure 1C). To explore the underlying mechanism that results in different prognoses, we performed the differentially expressed analysis and functional enrichment analyses between the clusters. As a result, 195 DEGs were identified, of which 106 were downregulated and 89 were upregulated (Figure 1D). GO analysis’s biological process (BP) was mainly enriched in cell adhesion, extracellular matrix organization, and cell differentiation. GO analysis’s cellular component (CC) was mainly enriched in the extracellular region, extracellular space, and extracellular exosome. GO analysis’s molecular function (MF) was mainly enriched in extracellular structural constituent, calcium-ion binding, and heparin binding (Figure 1E). KEGG analysis was mainly enriched in ECM–receptor interaction, protein digestion and absorption, focal adhesion, and the PI3K-Akt signaling pathway (Figure 1F). Furthermore, the GSEA was primarily enriched in glutathione metabolism, proteasome, oxidative phosphorylation, and fatty acid metabolism (Figure 1G). Therefore, the differences in cell adhesion and extracellular matrix metabolism of GO and KEGG enrichment analyses might affect OS invasiveness and metastasis ability, while metabolism-related pathways, such as glutathione metabolism, oxidative phosphorylation, and fatty acid metabolism, in GSEA may represent the different metabolism status among OS clusters, leading to the differential prognosis of OS patients. To sum up, m7G modification may be associated with extracellular matrix organization, focal adhesion, ECM–receptor interaction, and cell metabolism, which may affect the invasion and metabolism-related ability of OS cells, resulting in different prognoses among clusters.

**TABLE 1:** Prognostic genes generated using univariate Cox analysis.

Sig_genes	Full name	Category	Gene card ID	*p*-value	HR (95% Cl for HR)
*CYFIP2*	Cytoplasmic FMRl interacting protein 2	Protein coding	GC05P157267	0.0077	1.40 (1.10–1.80)
*IGF2BP2*	Insulin-like growth factor 2 MRNA-binding protein 2	Protein coding	GC03M185643	0.02	1.40 (1.00–1.90)
*ALKBHl*	AlkB homolog 1. Histone H2A dioxygenase	Protein coding	GC14M077672	0.019	0.31 (0.12–0.83)
*NUDTl*	Nudix hydrolase 1	Protein coding	GC07P002242	0.034	1.70 (1.00–2.70)
*FTO*	FTO alpha-ketoglutarate-dependent dioxygenase	Protein coding	GC16P053853	0.014	0.29 (0.11–0.78)
*EIF4Al*	Eukaryotic translation initiation factor 4Al	Protein coding	GC17P007572	0.021	2.50 (l.20–5.50)
*EIF4E3*	Eukaryotic translation initiation factor 4E family member 3	Protein coding	GC03M071675	0.014	0.31 (0.12–0.79)
*NUOT16*	Nudix hydrolase 16	Protein coding	GC03P131381	0.0019	0.30 (0.14–0.64)
*CYFIPl*	Cytoplasmic FMRl interacting protein 1	Protein coding	GC15M022867	0.0014	0.23 (0.09–0.56)

### Construction and validation of a prognostic model

**FIGURE 2:**
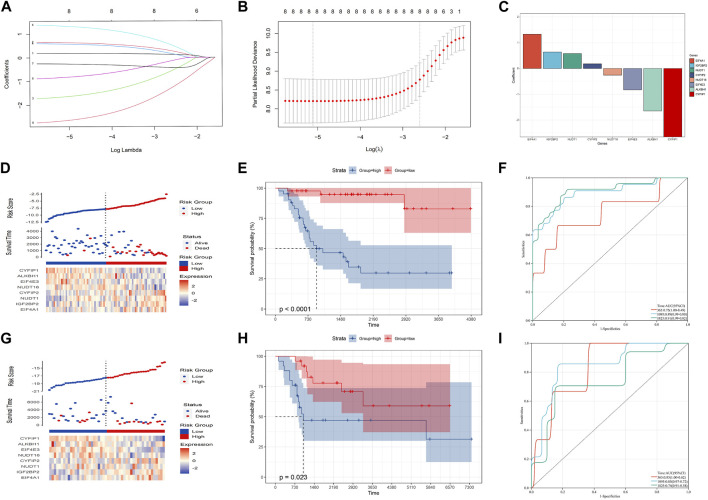
Construction of the prognostic model in the training cohort. **(A–C)** Eight candidate genes were screened out by LASSO analysis with minimal lambda. **(D–F)** Distribution, Kaplan–Meier plot, and time-dependent ROC curve of the risk model in the training group. **(G–I)** Distribution, Kaplan–Meier plot, and time-dependent ROC curve of the risk model in the testing group.

LASSO analysis was used to narrow down the prognostic m7G-related genes and construct a prognostic model based on the TARGET-OS dataset. Then, eight m7G-related genes were included in the final model, and the prognostic model could be expressed as follows: risk score = 0.18*CYFIP2 + 0.64*IGF2BP2 - 1.65*ALKBH1 + 0.58*NUDT1 + 1.33*EIF4A1 - 0.83*EIF4E3 - 0.26*NUDT16 - 2.66*CYFIP1 (Figures 2A–C). Subsequently, OS patients were separated into high- and low-risk groups based on the medium risk score, and the prognosis of the low-risk group was significantly better than that of the high-risk group (Figures 2D, E; *p*-value: <0.0001, HR: 13.01, 95CI% [3.89, 43.53]). The timeROC curve with the area under the curve (AUC) of 1-, 3-, and 5-year being 0.75, 0.89, and 0.91, respectively, signified the high prediction efficiency of the constructed prognostic model (Figure 2F). Meantime, the prognostic model’s predictive ability was tested in the GSE21257 dataset, suggesting the prognosis of the low-risk group was also better than that of the high-risk group. Moreover, the AUC of 1-, 3-, and 5-year ROC was 0.83, 0.85, and 0.74, respectively (Figures 2G–I; *p*-value: 0.023, HR: 2.82, 95CI% [1.11, 7.14]). Therefore, our prognostic model may exhibit excellent performance in predicting the prognosis of OS patients.

### Construction of an integrated nomogram

**FIGURE 3:**
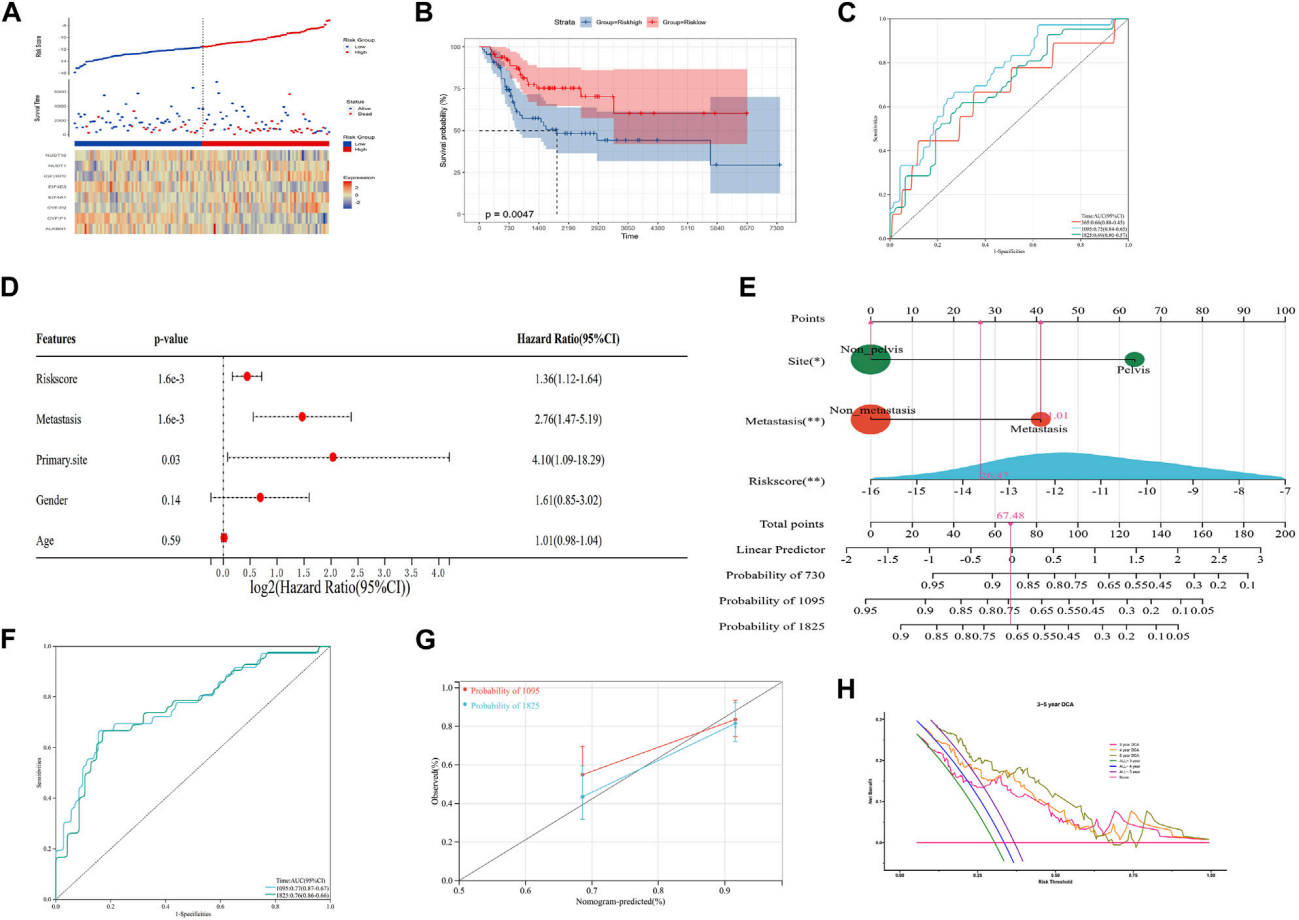
Construction of an integrated nomogram. **(A–C)** Distribution, Kaplan–Meier plot, and time-dependent ROC curve of the risk model in the merged group. ROC: receiver operating characteristic. **(D)** Result of multivariate Cox regression of the risk score and clinical characteristics. **(E)** Integrated nomogram combines the risk score and clinical characteristics, including metastasis and the tumor site. **(F, G)** timeROC curve, calibration curve, and timeDCA curve of the nomogram. ROC: receiver operating characteristic; DCA: decision curve analysis.

In order to improve the quantitative predictive ability of the risk model, we merged two cohorts (Supplementary Figure S2; Figures 3A–C; *p*-value: 0.0047, HR: 2.34, 95CI% [1.28, 4.28]) and integrated the clinical characteristics into the risk model to establish a nomogram. The result of multivariate Cox regression analysis indicated that the risk score (1.6 × 10^−3^), metastasis (1.6 × 10^−3^), and primary site (0.03) were the independent factors affecting the prognosis of OS patients, while the relationship between gender/age and the prognosis of OS patients was not significant (Figure 3D). Subsequently, we constructed an integrated nomogram including the risk score, metastasis, and primary site (Figure 3E). From the nomogram, each item can obtain its corresponding score and the total score and its corresponding survival rate can be obtained after adding the score of all items. The timeROC curve with a 3- and 5-year AUC being 0.77 and 0.76, respectively, indicated the predictive accuracy of the nomogram, and the results of the 3- and 5-year calibration plots confirmed this (Figures 3F, G). Furthermore, the 3-, 4-, and 5-year timeDCA curves indicated the excellent clinical usefulness of the nomogram (Figure 3H). Therefore, our integrated nomogram may serve as a viable quantitative predictor of the prognosis of OS patients.

### Mechanism exploration and functional enrichment analyses

**FIGURE 4:**
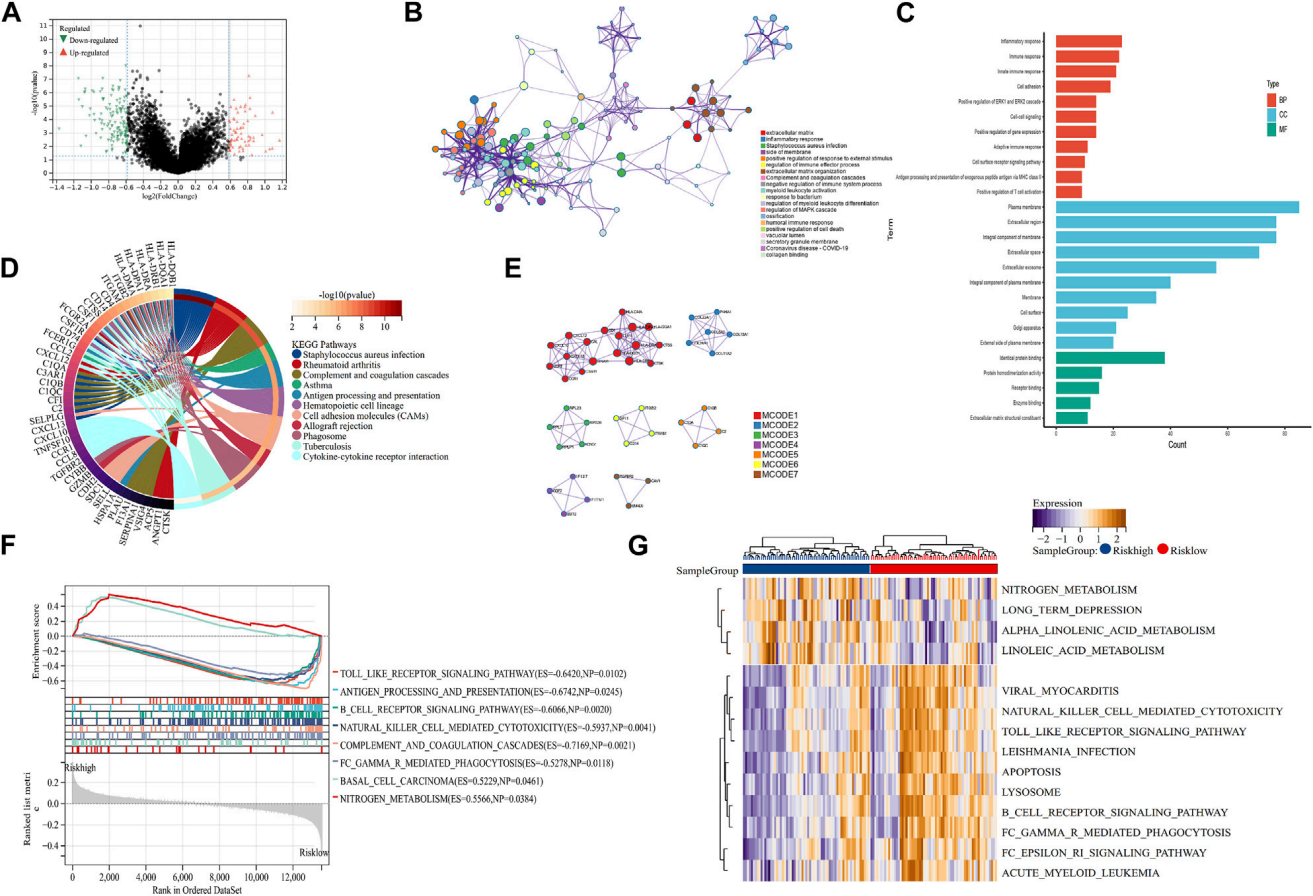
Mechanism exploration and functional enrichment analysis. **(A)** DEGs between the high- and low-risk groups. **(B, C)** PPI network and GO analysis of DEGs. **(D)** KEGG analysis of DEGs **(E)** Hub modules in the PPI network. **(F, G)** GSEA and GSVA of the high- and low-risk groups. DEGs: differentially expressed genes; PPI: protein–protein interaction; GO: Gene Ontology; KEGG: Kyoto Encyclopedia of Genes and Genomes; GSEA: Gene Set Enrichment Analysis; and GSVA: Gene Set Variation Analysis.

To explore the mechanism of our m7G-related prognostic model in OS, we performed differentially expressed analysis between the high- and low-risk groups. Then, 110 DEGs were identified, of which 69 were downregulated and 41 were upregulated (Figure 4A). GO function enrichment analysis indicated that DEGs were enriched in the immune-related processes and extracellular matrix metabolism (Figure 4B, C). Additionally, the KEGG enrichment analysis signified that DEGs were closely associated with some immune-related diseases and corresponding signaling pathways (Figure 4D). The PPI analysis further identified seven hub modules, which mainly involved the immune response and extracellular matrix metabolism (Figure 4E). Moreover, the GSEA and GSVA enrichment analyses were conducted to identify the expression pattern of the involved BP. The results revealed that immune-related processes, including antigen presentation, the B-cell receptor signaling pathway, and natural killer cell-mediated cytotoxicity, were highly expressed in the low-risk group, compared with the high-risk group. In contrast, the metabolism-related processes, including nitrogen metabolism, alpha linolenic acid metabolism, and linoleic acid metabolism, showed the opposite trend (Figure 4F, G). These function enrichment analyses synergistically suggested that m7G-related DEGs were closely associated with immunity disorders, extracellular matrix organization, and cellular metabolism in OS patients, which may be the underlying mechanism affecting the prognosis of OS patients.

### Immune infiltration analysis and drug sensitivity analysis

**FIGURE 5:**
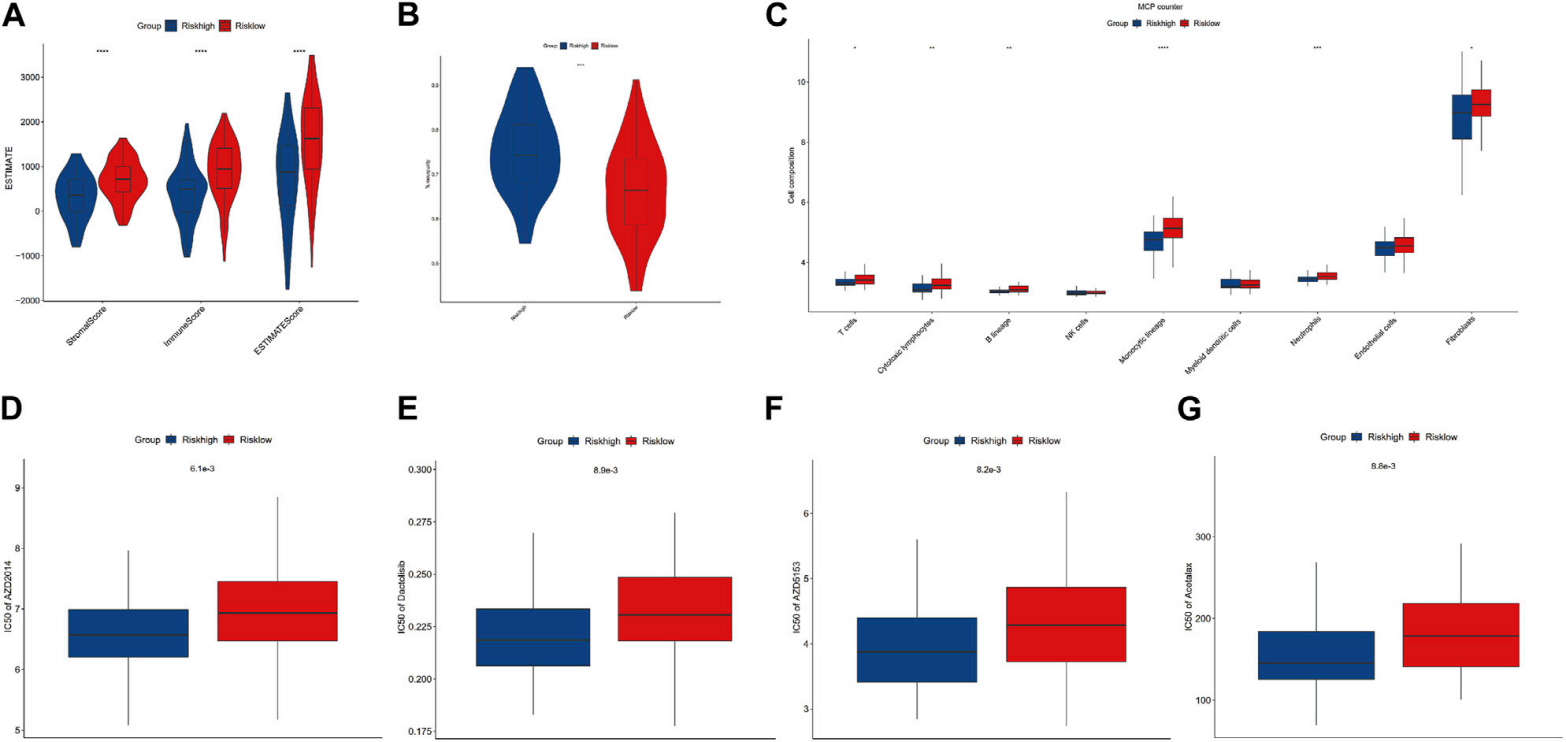
Immune infiltration and drug sensitivity analysis. **(A, B)** Comparisons between the high- and low-risk groups regarding stromal score, immune score, ESTIMATE score, and tumor purity. **(C)** MCP counter analysis. **(D–G)** Four potential drugs against OS.

To evaluate the impact of m7G modification on the OS immune microenvironment, we used ESTIMATE and MCP counter analyses to assess the infiltration of immune cells. Then, we found that the low-risk group’s stromal score, immune score, and estimate score were significantly higher. In contrast, the tumor purity of the low-risk group was significantly lower than that of the high-risk group (Figure 5A, B). Additionally, the MCP counter further confirmed the risk score was associated with the immune microenvironment and revealed the infiltration of T cells, cytotoxic lymphocytes, B lineage, monocytic lineage, neutrophils, and fibroblasts was distinctly higher in the low-risk group (Figure 5C). To determine the possible small molecules targeting m7G-related genes and further improve the clinical value of the prognostic model, we performed the drug sensitivity analysis by comparing IC50 between high- and low-risk groups. The results indicated that the high-risk group was more sensitive to AZD2014 (6.1×10^3^), AZD5153 (8.2×10^3^), acetalax (8.8×10^3^), and dactolisib (8.9×10^3^) in targeting m7G-related genes than the low-risk group (Figures 5D–G). AZD2014, the most significant sensitive drug in our drug sensitivity analysis, was included in the verified experiment.

### AZD2014 blocks OS cell division and induces apoptosis

**FIGURE 6:**
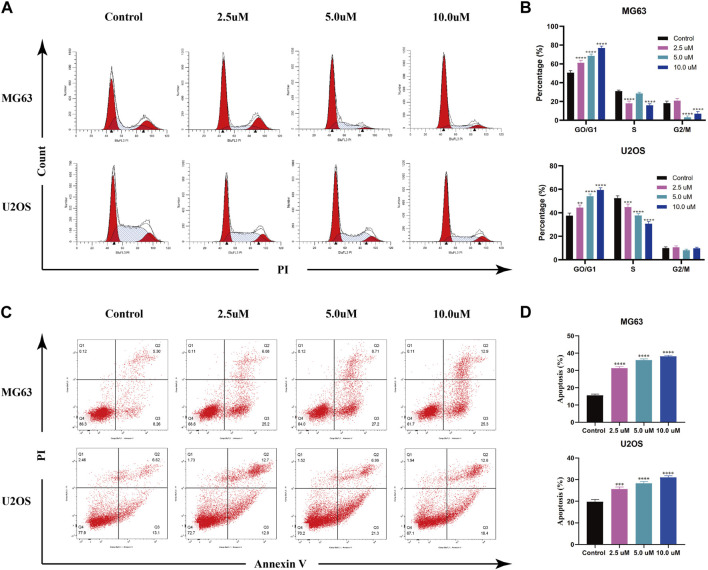
AZD2014 blocks OS cell division and induces apoptosis. **(A, B)** Impact of AZD2014 on the OS cell cycle; **(C, D)** impact of AZD2014 on OS cell apoptosis.

AZD 2014, an mTOR inhibitor, has been reported as an anti-proliferative drug against various cancers. To explore the impact of AZD2014 on OS, we performed the cell cycle assay and the apoptosis assay. The results of the cell cycle assays showed that the increase in AZD2014 expanded the proportion of G1-phase cells but decreased the proportion of S- and G2-phase cells, suggesting that AZD2014 can induce the G1-phase arrest in OS cells (Figure 6A, B). Subsequently, we analyzed the impact of AZD2014 on OS-cell apoptosis and found an increase of annexin V (+) OS cells followed by the high concentrations of AZD 2014, indicating that AZD2014 induced OS cell apoptosis as well (Figure 6C, D). As such, our results indicated that AZD2014 induced the G1-phase cell cycle arrest and apoptosis of OS cells, which may serve as a potential drug for OS.

### Verification of the m7G-related signature in OS

**FIGURE 7:**
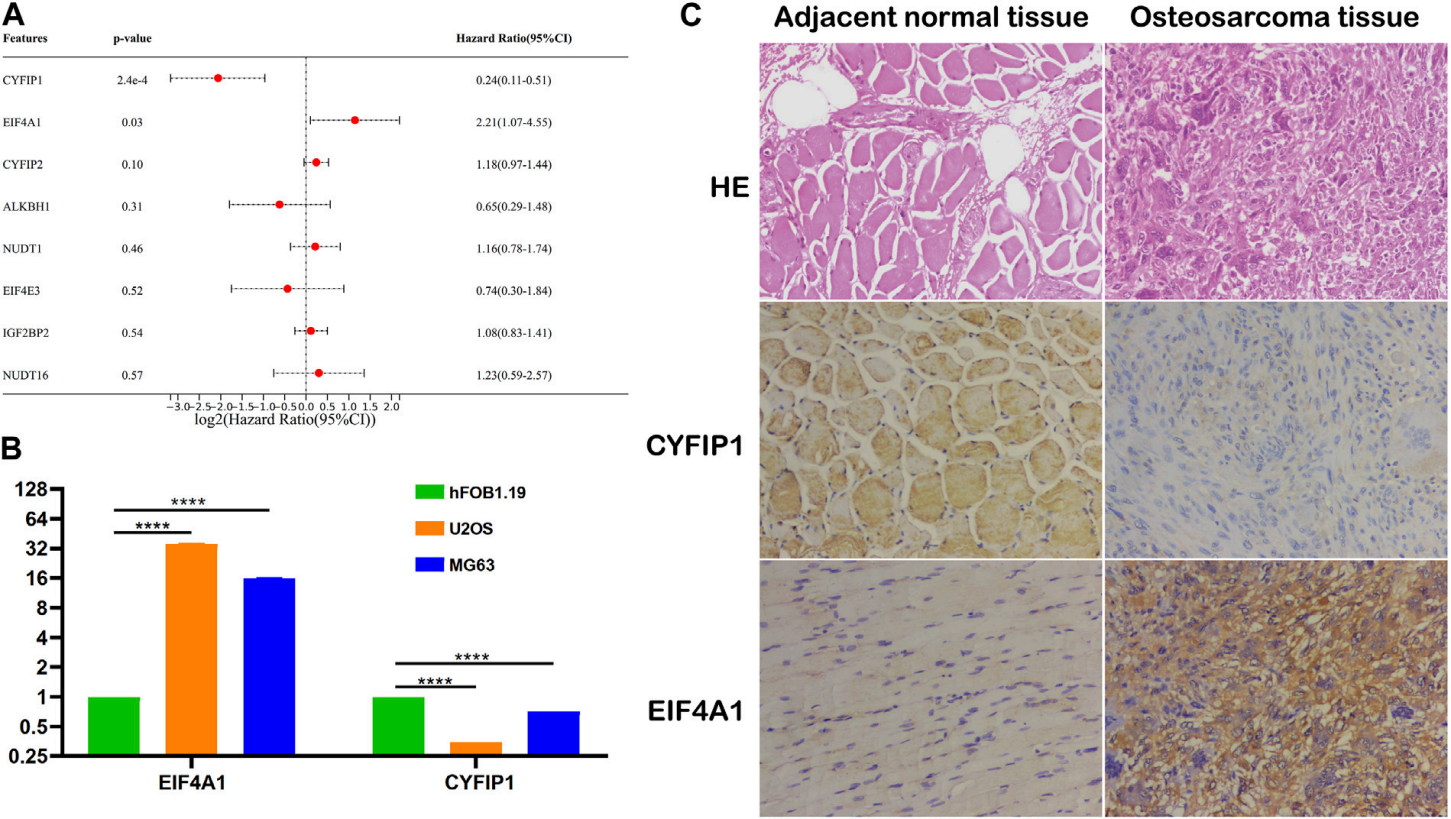
Validation of the m7G-related signatures. **(A)** Multivariate Cox regression analysis of the m7G-related signatures; **(B)** qPCR of CYFIP1 and EIF4A1 between osteoblasts and OS cells; and **(C)** IHC of CYFIP1 and EIF4A1 between OS tissue and adjacent normal tissue. qPCR: quantitative PCR; IHC: immunohistochemistry.

To explore the independent prognostic factors of OS, we performed multivariate Cox regression analysis among eight m7G-related signatures included in the prognostic model and found that *CYFIP1* and *EIF4A1* served as the independent prognostic factors of OS patients (Figure 7A). Subsequently, we carried out qPCR and IHC to validate our bioinformatic results. The result of qPCR illustrated that in comparison with osteoblasts, the expression of *CYFIP1* was significantly lower, while that of *EIF4A1* was higher in OS cells (Figure 7B). Meanwhile, IHC demonstrated that compared to the adjacent normal tissue, the expression of *CYFIP1* was significantly lower, while that of *EIF4A1* was evidently higher in OS tissue (Figure 7C). Taken together, our histological and cytological experiments validated the low expression of *CYFIP1* and high expression of *EIF4A1* in the OS status, which is consistent with our bioinformatic results, signifying the therapeutic potential of *CYFIP1* and *EIF4A1*.

## Discussion

Over the past decades, OS treatments have been stagnant because of the increase in chemotherapeutic resistance (Lin et al., 2021). Treatment advance requires further understanding of OS pathogenesis, progression, and drug resistance mechanism. Recently, m7G, a post-transcriptional modification, has been found to be involved in the oncogenesis, progression, and drug resistance of various cancers (Luo et al., 2022). Notably, previous studies have revealed the significance of m7G modification, such as *METTL1*-mediated tRNA modification, in driving oncogenic transformation and promoting resistance to specific drugs like lenvatinib (Orellana et al., 2021; Huang et al., 2023). Given the pivotal role of m7G modification in disease processes, researchers have focused on identifying and studying genes involved in the regulation of this modification to unravel their impact during various disease states (Huang et al., 2022a; Li X. Y. et al., 2022). Similarly, our study aimed to employ bioinformatic analysis to explore the prognostic significance of m7G-related genes in OS patients. Through univariate Cox and LASSO regression analyses, we identified eight m7G-related genes, including *EIF4A1*, *IGF2BP2*, *NUDT1*, *CYFIP2*, *NUDT16*, *EIF4E3*, *ALKBH1*, and *CYFIP1,* and developed a prognostic model based on their expression patterns. Subsequently, we calculated a corresponding risk score using these m7G-related genes, which could potentially serve as a valuable prognostic and chemosensitive predictive tool for OS. Furthermore, we investigated the impact of AZD 2014, a drug of interest, on the cell cycle and apoptosis of OS cells. Our findings demonstrated that AZD2014 induces the G1-phase cell cycle arrest and apoptosis in OS cells. These observations suggest that the identified m7G-related genes may not only serve as indicators of drug sensitivity but also have clinical implications for predicting the outcomes of OS patients.

With the increasing understanding of genetics, epigenetic regulation has obtained significant attention in various biological and pathological processes (McKusick, 1970). Among the crucial RNA modifications involved in epigenetic regulation, m7G modification has emerged as a subject of growing interest (Dai et al., 2021). Numerous studies have demonstrated the wide-ranging involvement of m7G modification in the pathogenesis and development of various diseases, particularly cancers (Chen Y. et al., 2022). For example, overactive *METTL1* can promote the methylation and maturation of m7G in let-7 miRNA, a tumor suppressor miRNA, thereby inhibiting the metastasis of lung cancer cells (Pandolfini et al., 2019). Conversely, the disruption of m7G modification resulting from *METTL1* knockout leads to global translation defects of oncogenes and the loss of typical malignant transformation markers, thereby inhibiting the occurrence and development of intrahepatic cholangiocarcinoma (Dai et al., 2021). However, unlike other tumor types, the study of m7G modification in OS remains largely unexplored. In this study, we focused on clustering OS patients based on the prognostic value of m7G-related genes determined by univariate Cox regression analysis. Our findings revealed a significant association between m7G modification and the prognosis of OS patients. To quantitatively assess the impact of m7G modification on OS patients, we constructed a prognostic model. The discriminatory power of this model was validated in both training and testing groups, and its performance was further evaluated in the merged group using timeROC curves. Furthermore, the independence of the prognostic model was demonstrated through multivariate Cox regression analysis in the merged cohort. Additionally, we identified metastasis and the primary tumor site as independent factors influencing the prognosis of OS patients. To enhance the accuracy of prognosis prediction in OS patients, we developed a nomogram that integrates the risk score derived from the prognostic model with clinical characteristics, including metastasis and primary sites. The calibration plots for the 3- and 5-year outcomes, as well as the timeROC curves, demonstrated the efficacy and accuracy of the nomogram in predicting patient prognosis. Furthermore, the timeDCA curve indicated the excellent clinical utility of this nomogram, further supporting its potential as a valuable predictive tool for clinicians. Consequently, our study highlights the importance of m7G modification in OS and presents a robust prognostic model and nomogram that incorporate m7G-related genes and clinical characteristics for accurate prognostic prediction. The findings underscore the potential clinical usefulness of this approach in guiding treatment decisions and improving patient outcomes in OS.

Notably, our prognostic model revealed an interesting association between the expression of m7G-related genes in OS patients and their immune status. Patients with better prognoses displayed more active immune statuses, suggesting a potential correlation between m7G modification and immune responses in OS. In recent years, increasing evidence has emphasized the intricate interplay and coevolution between tumor cells, immune components, and the tumor stroma, underscoring their significant roles in cancer pathogenesis, progression, and treatment (Gill and Gorlick, 2021; Heymann et al., 2021; Huang et al., 2022b; Huang R. et al., 2022). Previous studies have highlighted the involvement of tumor stroma in various processes, including neovascularization, inherent features for tumor homing, microvesicle secretion, paracrine cross-feeding, and immune modulation, all contributing toward tumor progression (Cortini et al., 2017). Additionally, immune cells, such as myeloid-derived suppressor cells, regulatory T cells, and tumor-associated macrophages, have been identified as key players in regulating tumorigenesis and tumor growth (Xia et al., 2022). To explore the impact of m7G modifications on the TME, we used ESTIMATE and MCP counter analyses. The results confirmed that the low-risk group, as defined by our m7G-related risk scores, exhibited higher levels of immune infiltration compared to the high-risk group. Furthermore, functional enrichment analysis of DEGs and PPI network hub modules provided additional evidence that the immune score of the low-risk group was higher than that of the high-risk group. Collectively, these findings suggested that m7G-related risk scores could serve as a valuable reference for guiding immunotherapeutic strategies in OS. Therefore, our findings underscored the significance of considering m7G-related gene expressions as potential indicators for immunotherapies, further emphasizing the interplay between epigenetic modifications, immune responses, and OS pathogenesis.

The emergence of chemotherapeutic resistance and the propensity for metastasis and recurrence pose significant challenges in the treatment of OS, necessitating the exploration of novel therapeutic options. In our research, we identified four potential drugs that hold promise for OS treatment: AZD 2014, AZD5153, acetalax, and dactolisib. Previous studies have demonstrated the anti-tumor effects of AZD5153, acetalax, and dactolisib in OS (Gobin et al., 2014; Sun et al., 2020; Sheard et al., 2021). However, the impact of AZD2014 on OS remains to be elucidated. AZD 2014, also known as vistusertib, is an ATP-competitive *mTOR1/2* inhibitor with broad-spectrum anti-cancer properties, exhibiting a strong anti-proliferative activity (Zheng et al., 2015; Pi et al., 2021). The *PI3K/Akt/mTOR* signaling pathway has been implicated in the occurrence and development of OS, suggesting that targeting this pathway may hold promise for OS treatment (Gill and Gorlick, 2021). Additionally, recent studies have indicated a link between m7G modifications and the *mTOR* pathway in the pathogenesis and progression of certain cancers (Chen J. et al., 2022; Han et al., 2023). In our study, we demonstrated that AZD2014 induces the G1-phase cell cycle arrest and apoptosis in OS cells through cell cycles and apoptosis assays. These findings are consistent with the results obtained by Pi et al. (2021), who observed similar effects of AZD2014 in ovarian cancer. Collectively, our results suggested that AZD2014 may represent a promising therapeutic option for OS treatment, warranting further investigation.

Finally, our study identified *CYFIP1* and *EIF4A1* as two independent risk factors for OS through multivariate Cox regression analysis. We further confirmed the differential expression of *CYFIP1* and *EIF4A1* at both histological and cytological levels. *CYFIP1,* also known as the *SRA1* protein*,* is a component of the *CYFIP1–EIF4E–FMR1* complex, which binds to the mRNA cap and regulates translational repression (De Rubeis et al., 2013). Recent studies have demonstrated the significance of *CYFIP1* in cancer development, particularly in promoting invasion (Silva et al., 2009). *EIF4A1* is an ATP-dependent RNA helicase that participates in the assembly of the *EIF4F* complex that is essential for cap recognition and mRNA binding to ribosomes (Schmidt et al., 2023). During protein translation, *EIF4A1* unwinds RNA secondary structures in the 5′-UTR of mRNA, facilitating the efficient binding of small ribosomal subunits and subsequent scanning for initiator codons (Tauber et al., 2020). Increasing evidence has highlighted the oncogenic role of *EIF4A1* in various cancers, including prostate, pancreatic, and breast cancer (Modelska et al., 2015; Ma et al., 2019; Wang et al., 2022). Given the significant involvement of *CYFIP1* and *EIF4A1* in cancer pathogenesis, including their potential roles in invasion and protein translation, targeting these molecules may hold promise as a therapeutic approach for OS. Developing therapies that specifically modulate the functions of *CYFIP1* and *EIF4A1* could provide new avenues for improving the treatment outcomes of OS patients.

Undoubtedly, there are several limitations that should be acknowledged in the present study. First, the rare incidence of OS poses challenges in recruiting a large cohort for comprehensive analysis, which may limit the generalizability of the findings. Future studies should aim to collaborate with multiple research centers or utilize international databases to overcome this limitation. Second, it is important to acknowledge that the RNA-seq and clinical data utilized in this study were predominantly sourced from TCGA and GEO databases, which primarily represent European and North American populations. This may introduce inherent selection bias and limit the applicability of the findings to other ethnic populations (Tomczak et al., 2015). Therefore, it is crucial to validate the results in diverse cohorts that encompass different ethnic backgrounds and geographical regions. Furthermore, the bioinformatic analysis performed in this study relies on publicly available databases, which may contain inherent limitations and inconsistencies. The accuracy and reliability of the results are contingent on the quality and comprehensiveness of the data sources. Therefore, caution should be exercised in the interpretation and application of the findings. Last, the current study is based on bioinformatic analysis and *in vitro* experiments, which provide valuable insights into the role of m7G-related genes in OS. However, further *in vivo* experiments and clinical studies are warranted to validate the findings and assess the clinical feasibility of the identified m7G-related risk model and potential therapeutic targets.

## Conclusion

In summary, our study sheds light on the potential importance of m7G-related genes in OS. Through the establishment of a prognostic model and investigation of drug responses, we obtained valuable insights into the role of m7G modification in the pathogenesis of OS and its impact on treatment outcomes. Particularly, our findings suggest that AZD2014 holds promise as a potential therapeutic agent for OS. Moreover, we identified *CYFIP1* and *EIF4A1* as independent risk factors for OS. These findings contribute to a better understanding of the molecular mechanisms underlying OS and may pave the way for the development of targeted therapies. Further research and clinical validation are warranted to explore the full therapeutic potential of m7G-related genes in OS.

## Data Availability

The original contributions presented in the study are included in the article/Supplementary Material; further inquiries can be directed to the corresponding author.

## References

[B1] Aljubran A. H. Griffin A. Pintilie M. Blackstein M. (2009). Osteosarcoma in adolescents and adults: survival analysis with and without lung metastases. Ann. Oncol. 20, 1136–1141. 10.1093/annonc/mdn731 19153114

[B2] Chen J. Li K. Wang X. Ling R. Cheng M. (2022b). Aberrant translation regulated by METTL1/WDR4-mediated tRNA N7-methylguanosine modification drives head and neck squamous cell carcinoma progression. Cancer Commun. Lond. Engl. 42, 223–244. 10.1002/cac2.12273 PMC8923133 35179319

[B3] Chen Y. Lin H. Miao L. He J. (2022a). Role of N7-methylguanosine (m^7^G) in cancer. Trends Cell Biol. 32, 819–824. 10.1016/j.tcb.2022.07.001 35907701

[B4] Chen Z. Zhu W. Zhu S. Sun K. Liao J. Liu H. (2021). METTL1 promotes hepatocarcinogenesis via m(7) G tRNA modification-dependent translation control. Clin. Transl. Med. 11, e661. 10.1002/ctm2.661 34898034 PMC8666584

[B5] Cortini M. Avnet S. Baldini N. (2017). Mesenchymal stroma: role in osteosarcoma progression. Cancer Lett. 405, 90–99. 10.1016/j.canlet.2017.07.024 28774797

[B6] Dai Z. Liu H. Liao J. Huang C. Ren X. Zhu W. (2021). N(7)-Methylguanosine tRNA modification enhances oncogenic mRNA translation and promotes intrahepatic cholangiocarcinoma progression. Mol. Cell 81, 3339–3355.e8. 10.1016/j.molcel.2021.07.003 34352206

[B7] De Rubeis S. Pasciuto E. Li K. W. Fernández E. Di Marino D. Buzzi A. (2013). CYFIP1 coordinates mRNA translation and cytoskeleton remodeling to ensure proper dendritic spine formation. Neuron 79, 1169–1182. 10.1016/j.neuron.2013.06.039 24050404 PMC3781321

[B8] Dong K. Gu D. Shi J. Bao Y. Fu Z. Fang Y. (2022). Identification and verification of m(7)G modification patterns and characterization of tumor microenvironment infiltration via multi-omics analysis in clear cell renal cell carcinoma. Front. Immunol. 13, 874792. 10.3389/fimmu.2022.874792 35592316 PMC9113293

[B9] Gao Z. Xu J. Zhang Z. Fan Y. Xue H. Guo X. (2022). A comprehensive analysis of METTL1 to immunity and stemness in pan-cancer. Front. Immunol. 13, 795240. 10.3389/fimmu.2022.795240 35432338 PMC9008260

[B10] Gill J. Gorlick R. (2021). Advancing therapy for osteosarcoma. Nat. Rev. Clin. Oncol. 18, 609–624. 10.1038/s41571-021-00519-8 34131316

[B11] Gobin B. Battaglia S. Lanel R. Chesneau J. Amiaud J. Rédini F. (2014). NVP-BEZ235, a dual PI3K/mTOR inhibitor, inhibits osteosarcoma cell proliferation and tumor development *in vivo* with an improved survival rate. Cancer Lett. 344, 291–298. 10.1016/j.canlet.2013.11.017 24333720

[B12] Guy M. P. Phizicky E. M. (2014). Two-subunit enzymes involved in eukaryotic post-transcriptional tRNA modification. RNA Biol. 11, 1608–1618. 10.1080/15476286.2015.1008360 25625329 PMC4615748

[B13] Han H. Yang C. Ma J. Zhang S. Zheng S. Ling R. (2022). N(7)-methylguanosine tRNA modification promotes esophageal squamous cell carcinoma tumorigenesis via the RPTOR/ULK1/autophagy axis. Nat. Commun. 13, 1478. 10.1038/s41467-022-29125-7 35304469 PMC8933395

[B14] Han H. Zheng S. Lin S. (2023). N(7)-methylguanosine (m(7)G) tRNA modification: a novel autophagy modulator in cancer. Autophagy 19, 360–362. 10.1080/15548627.2022.2077551 35574843 PMC9809925

[B15] Heymann M. F. Schiavone K. Heymann D. (2021). Bone sarcomas in the immunotherapy era. Br. J. Pharmacol. 178, 1955–1972. 10.1111/bph.14999 31975481

[B16] Huang M. Long J. Yao Z. Zhao Y. Zhao Y. Liao J. (2023). METTL1-Mediated m7G tRNA modification promotes Lenvatinib resistance in hepatocellular carcinoma. Cancer Res. 83, 89–102. 10.1158/0008-5472.Can-22-0963 36102722

[B17] Huang R. Wang X. Yin X. Zhou Y. Sun J. Yin Z. (2022c). Combining bulk RNA-sequencing and single-cell RNA-sequencing data to reveal the immune microenvironment and metabolic pattern of osteosarcoma. Front. Genet. 13, 976990. 10.3389/fgene.2022.976990 36338972 PMC9626532

[B18] Huang X. Chen Z. Xiang X. Liu Y. Long X. Li K. (2022a). Comprehensive multi-omics analysis of the m7G in pan-cancer from the perspective of predictive, preventive, and personalized medicine. EPMA J. 13, 671–697. 10.1007/s13167-022-00305-1 36505892 PMC9727047

[B19] Huang X. Wang L. Guo H. Zhang W. Shao Z. (2022b). Single-cell transcriptomics reveals the regulative roles of cancer associated fibroblasts in tumor immune microenvironment of recurrent osteosarcoma. Theranostics 12, 5877–5887. 10.7150/thno.73714 35966586 PMC9373820

[B20] Li L. Yang Y. Wang Z. Xu C. Huang J. (2021). Prognostic role of METTL1 in glioma. Cancer Cell Int. 21, 633. 10.1186/s12935-021-02346-4 34838021 PMC8627054

[B21] Li X. Y. Zhao Z. J. Wang J. B. Shao Y. H. Hui L. You J. X. (2022b). m7G methylation-related genes as biomarkers for predicting overall survival outcomes for hepatocellular carcinoma. Front. Bioeng. Biotechnol. 10, 849756. 10.3389/fbioe.2022.849756 35620469 PMC9127183

[B22] Li Z. Li Y. Shen L. Shen L. Li N. (2022a). Molecular characterization, clinical relevance and immune feature of m7G regulator genes across 33 cancer types. Front. Genet. 13, 981567. 10.3389/fgene.2022.981567 36092891 PMC9453236

[B23] Lin Z. Xie X. Lu S. Liu T. (2021). Noncoding RNAs in osteosarcoma: implications for drug resistance. Cancer Lett. 504, 91–103. 10.1016/j.canlet.2021.02.007 33587978

[B24] Link M. P. Goorin A. M. Miser A. W. Green A. A. Pratt C. B. Belasco J. B. (1986). The effect of adjuvant chemotherapy on relapse-free survival in patients with osteosarcoma of the extremity. N. Engl. J. Med. 314, 1600–1606. 10.1056/nejm198606193142502 3520317

[B25] Luo Y. Yao Y. Wu P. Zi X. Sun N. He J. (2022). The potential role of N(7)-methylguanosine (m7G) in cancer. J. Hematol. Oncol. 15, 63. 10.1186/s13045-022-01285-5 35590385 PMC9118743

[B26] Ma X. Li B. Liu J. Fu Y. Luo Y. (2019). Phosphoglycerate dehydrogenase promotes pancreatic cancer development by interacting with eIF4A1 and eIF4E. J. Exp. Clin. cancer Res. CR 38, 66. 10.1186/s13046-019-1053-y 30744688 PMC6371491

[B27] McKusick V. A. (1970). Human genetics. Annu. Rev. Genet. 4, 1–46. 10.1146/annurev.ge.04.120170.000245 4950059

[B28] Meltzer P. S. Helman L. J. (2021). New horizons in the treatment of osteosarcoma. N. Engl. J. Med. 385, 2066–2076. 10.1056/NEJMra2103423 34818481

[B29] Meyers P. A. Healey J. H. Chou A. J. Wexler L. H. Merola P. R. Morris C. D. (2011). Addition of pamidronate to chemotherapy for the treatment of osteosarcoma. Cancer 117, 1736–1744. 10.1002/cncr.25744 21472721 PMC3059356

[B30] Modelska A. Turro E. Russell R. Beaton J. Sbarrato T. Spriggs K. (2015). The malignant phenotype in breast cancer is driven by eIF4A1-mediated changes in the translational landscape. Cell death Dis. 6, e1603. 10.1038/cddis.2014.542 25611378 PMC4669741

[B31] Okamoto M. Fujiwara M. Hori M. Okada K. Yazama F. Konishi H. (2014). tRNA modifying enzymes, NSUN2 and METTL1, determine sensitivity to 5-fluorouracil in HeLa cells. PLoS Genet. 10, e1004639. 10.1371/journal.pgen.1004639 25233213 PMC4169382

[B32] Orellana E. A. Liu Q. Yankova E. Pirouz M. De Braekeleer E. Zhang W. (2021). METTL1-mediated m(7)G modification of Arg-TCT tRNA drives oncogenic transformation. Mol. Cell 81, 3323–3338.e14. 10.1016/j.molcel.2021.06.031 34352207 PMC8380730

[B33] Pandolfini L. Barbieri I. Bannister A. J. Hendrick A. Andrews B. Webster N. (2019). METTL1 promotes let-7 MicroRNA processing via m7G methylation. Mol. Cell 74, 1278–1290. 10.1016/j.molcel.2019.03.040 31031083 PMC6591002

[B34] Pi R. Yang Y. Hu X. Li H. Shi H. Liu Y. (2021). Dual mTORC1/2 inhibitor AZD2014 diminishes myeloid-derived suppressor cells accumulation in ovarian cancer and delays tumor growth. Cancer Lett. 523, 72–81. 10.1016/j.canlet.2021.09.017 34560229

[B35] Regmi P. He Z. Q. Lia T. Paudyal A. Li F. Y. (2022). N7-Methylguanosine genes related prognostic biomarker in hepatocellular carcinoma. Front. Genet. 13, 918983. 10.3389/fgene.2022.918983 35734429 PMC9207530

[B36] Ritter J. Bielack S. S. (2010). Osteosarcoma. Ann. Oncol. 21 (7), vii320–325. 10.1093/annonc/mdq276 20943636

[B37] Schmidt T. Dabrowska A. Waldron J. A. Hodge K. Koulouras G. Gabrielsen M. (2023). eIF4A1-dependent mRNAs employ purine-rich 5'UTR sequences to activate localised eIF4A1-unwinding through eIF4A1-multimerisation to facilitate translation. Nucleic acids Res. 51, 1859–1879. 10.1093/nar/gkad030 36727461 PMC9976904

[B38] Sheard J. J. Southam A. D. Mackay H. L. Ellington M. A. Snow M. D. Khanim F. (2021). Combined bezafibrate, medroxyprogesterone acetate and valproic acid treatment inhibits osteosarcoma cell growth without adversely affecting normal mesenchymal stem cells. Biosci. Rep. 41. 10.1042/bsr20202505 PMC7786328 33289496

[B39] Silva J. M. Ezhkova E. Heart S. Castillo M. Campos Y. (2009). Cyfip1 is a putative invasion suppressor in epithelial cancers. Cell 137, 1047–1061. 10.1016/j.cell.2009.04.013 19524508 PMC2754270

[B40] Sloan K. E. Warda A. S. Sharma S. Entian K. D. Lafontaine D. L. J. Bohnsack M. T. (2017). Tuning the ribosome: the influence of rRNA modification on eukaryotic ribosome biogenesis and function. RNA Biol. 14, 1138–1152. 10.1080/15476286.2016.1259781 27911188 PMC5699541

[B41] Song B. Tang Y. Chen K. Wei Z. Rong R. Lu Z. (2020). m7GHub: deciphering the location, regulation and pathogenesis of internal mRNA N7-methylguanosine (m7G) sites in human. Bioinforma. Oxf. Engl. 36, 3528–3536. 10.1093/bioinformatics/btaa178 32163126

[B42] Sun Y. Han J. Wang Z. Li X. Hu Z. (2020). Safety and efficacy of bromodomain and extra-terminal inhibitors for the treatment of hematological malignancies and solid tumors: a systematic study of clinical trials. Front. Pharmacol. 11, 621093. 10.3389/fphar.2020.621093 33574760 PMC7870522

[B43] Tauber D. Tauber G. Khong A. Van Treeck B. Pelletier J. Parker R. (2020). Modulation of RNA condensation by the DEAD-box protein eIF4A. Cell 180, 411–426. 10.1016/j.cell.2019.12.031 31928844 PMC7194247

[B44] Tian Q. H. Zhang M. F. Zeng J. S. Luo R. G. Wen Y. Chen J. (2019). METTL1 overexpression is correlated with poor prognosis and promotes hepatocellular carcinoma via PTEN. J. Mol. Med. (Berlin, Ger. 97, 1535–1545. 10.1007/s00109-019-01830-9 31463732

[B45] Tomczak K. Czerwińska P. Wiznerowicz M. (2015). The cancer genome atlas (TCGA): an immeasurable source of knowledge. Contemp. Oncol. (Poznan, Pol. 19, A68–A77. 10.5114/wo.2014.47136 PMC4322527 25691825

[B46] Wang C. Leavenworth J. Zhang C. Liu Z. Yuan K. Y. Wang Y. (2022). Epigenetic regulation of EIF4A1 through DNA methylation and an oncogenic role of eIF4A1 through BRD2 signaling in prostate cancer. Oncogene 41, 2778–2785. 10.1038/s41388-022-02272-3 35361883 PMC9215223

[B47] Wang Y. T. Chen J. Chang C. W. Jen J. Huang T. Y. Chen C. M. (2017). Ubiquitination of tumor suppressor PML regulates prometastatic and immunosuppressive tumor microenvironment. J. Clin. investigation 127, 2982–2997. 10.1172/jci89957 PMC5531412 28691927

[B48] Wu X. Li C. Wang Z. Zhang Y. Liu S. Chen S. (2022). A bioinformatic analysis study of m(7)G regulator-mediated methylation modification patterns and tumor microenvironment infiltration in glioblastoma. BMC cancer 22, 729. 10.1186/s12885-022-09791-y 35788194 PMC9251941

[B49] Wylie J. (2004). Pathology and genetics of tumours of soft tissue and bone. Published 2002, 1st edition, ISBN 92 832 24132. Surg. Oncol. 13, 43. 10.1016/j.suronc.2004.03.001

[B50] Xia Y. Wang D. Piao Y. Chen M. Wang D. Jiang Z. (2022). Modulation of immunosuppressive cells and noncoding RNAs as immunotherapy in osteosarcoma. Front. Immunol. 13, 1025532. 10.3389/fimmu.2022.1025532 36457998 PMC9705758

[B51] Zeng X. Liao G. Li S. Liu H. Zhao X. Li S. (2023). Eliminating METTL1-mediated accumulation of PMN-MDSCs prevents hepatocellular carcinoma recurrence after radiofrequency ablation. Hepatol. Baltim. Md.) 77, 1122–1138. 10.1002/hep.32585 35598182

[B52] Zheng B. Mao J. H. Qian L. Zhu H. Gu D. h. Pan X. d. (2015). Pre-clinical evaluation of AZD-2014, a novel mTORC1/2 dual inhibitor, against renal cell carcinoma. Cancer Lett. 357, 468–475. 10.1016/j.canlet.2014.11.012 25444920

