# Supplementary material for: m7G-related genes predict prognosis and affect the immune microenvironment and drug sensitivity in osteosarcoma
Source: Front Pharmacol. 2023 Aug 16;14:1158775. doi: 10.3389/fphar.2023.1158775 (PMC10466804; doi:10.3389/fphar.2023.1158775)
Supplement: Supplementary file 1 [file Image1.pdf]

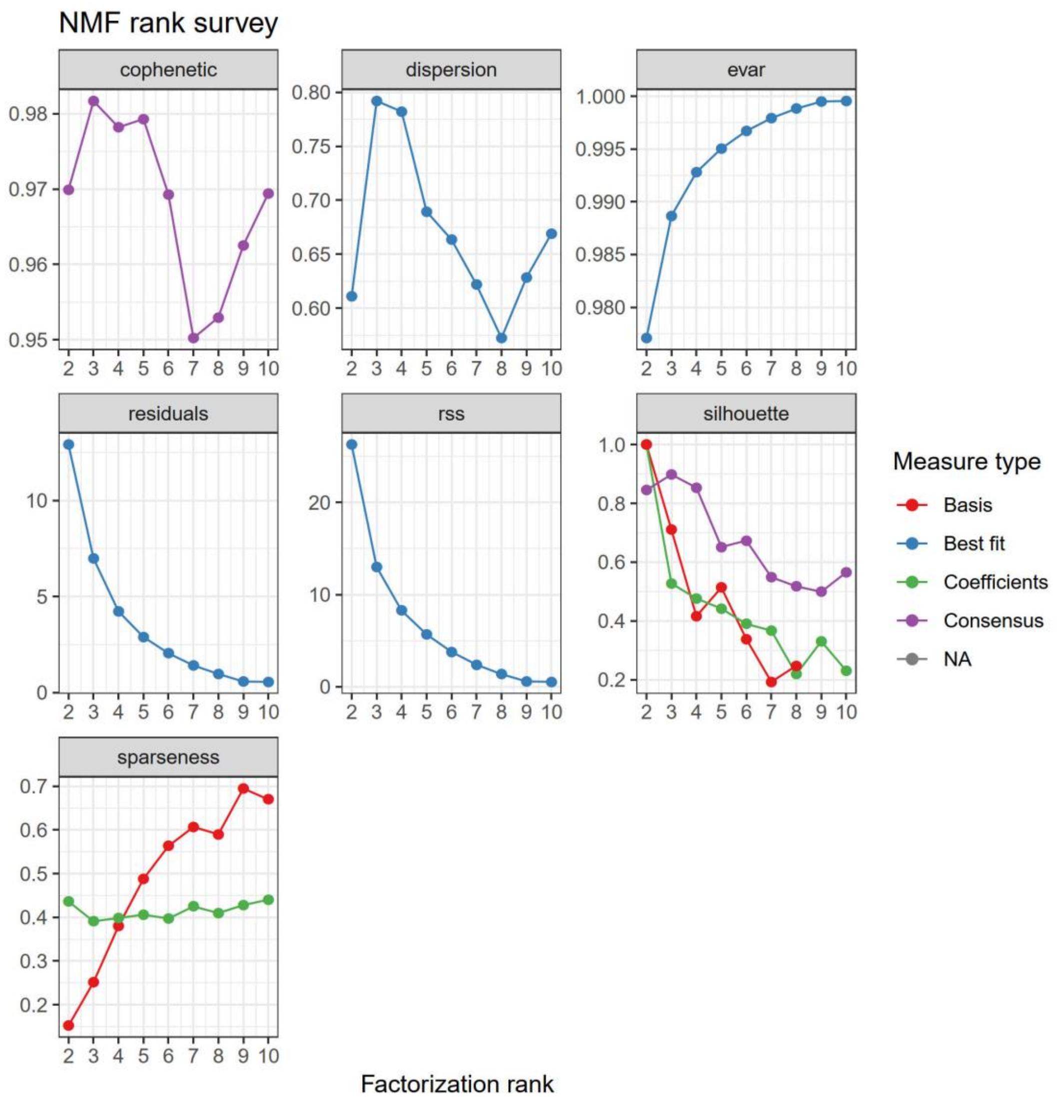

Supplementary figure1. The cophenetic plot for choosing the best clustering

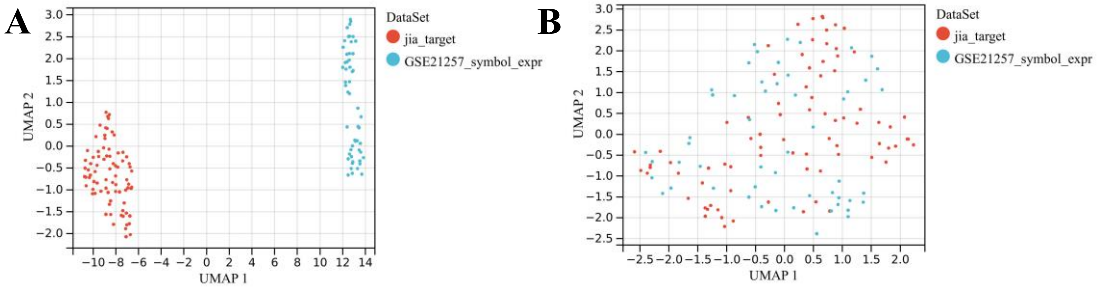

Supplementary figure2. (A) Before merging (B) After merging
